# Effect of Nano Alumina on the Properties of Fluorinated Polyurethane

**DOI:** 10.3390/ma12244120

**Published:** 2019-12-09

**Authors:** Ruizhu Zhang, Wenbo Wang, Chongyang Wang, Wejie Tian, Jianlin Hang, M. Irfan Hussain

**Affiliations:** 1 School of Mechanical Engineering, North China University of Water Resources and Electric Power, Zhengzhou 450045, Chinawcy199436@163.com (C.W.); tian102623@163.com (W.T.); 2 Henan Tianma New Material Co., Ltd., Zhengzhou 450025, China; zztmwfgs158@sina.com; 3 School of Materials Science and Engineering, University of Science and Technology Beijing, Beijing 100083, China; h.irfan.uaf@Outlook.com

**Keywords:** fluorinated polyurethane, nano-alumina, coating, water resistant, anti-wear

## Abstract

This article selects studies on the preparation of fluorinated polyurethane-nano-alumina composite coating materials, and analyzes the anti-wear, water resistantance, and surface microstructure. Attenuated total reflection-Fourier transform infrared spectroscopy (ATR-FTIR) shows that the polyurethane synthesized in this study does not contain hydrophilic –CH_2_OH groups. The cavitation wear test depicts that the actual cavitation amount C of the Al_2_O_3_-FPU (4) (fluorinated polyurethane) coating is 0.9035 × 10^−3^ kg, and the anti-wear ability increases by 61.9% compared with FPU-0.5. The water-resistant test shows that the contact angle of water droplets on the surface of the coating increase from 95.3° of FPU-0.5 to 123.1° of Al_2_O_3_-FPU (4), and the water absorption decreases from 2.52% to 1.04%. Scanning electron microscopy (SEM) observation confirms that alumina particles can protrude on the coating surface and resist strong wear, while the C-F chain with high bond energy at the near-surface exhibits high strength and water resistance, which prevents wear from spreading deep into the coating. Differential scanning calorimetry (DSC) results show that the Tg_(HS)_ value of the hard segment phase decreases with higher external force. Notably, when the coating is subjected to erosion, which enhances the crystallinity of the hard segment phase, the tensile strength of the hard segment phase of the coating surface is improved, which supports the wear resistance. Herein, we show that the addition of nano-alumina to fluorinated polyurethanes can control high water and abrasion resistance.

## 1. Introduction

Surface damage and destruction caused by cavitation wear have always been a great challenge during operating and maintenance management of hydraulic engineering [1]. Yet, most wear-resistant and anti-corrosive coatings contain organic volatiles that are extremely harmful to the environment and water resources. Besides, ordinary epoxy coatings are not suitable for applications with impact protection and elastic expansion because they are too hard and insufficiently flexibility. Polyurethane is a polymer material with excellent physical properties, namely, high elasticity, high tear resistance, wear resistance, and anti-corrosion [2,3,4]. Even though there has been continuous development and improvement of polyurethane, its application range still faces some serious challenges and needs to be made more extensive. However, due to its good anti-fatigue, cavitation resistance, and high- and low-temperature toughness, it plays an important role in the anti-wear protection of underwater concrete and hydraulic engineering steel structures [5,6,7]. Shuang Xiao et al. [8] studied the effect of molecular weight on the scratch and abrasive wear behavior of three model thermoplastic polyurethane (TPU) elastomers. Studies have found that increasing the molecular weight can increase the tensile strength of TPU samples. Higher molecular weights have enhanced resistance to cracking and scratching. However, the limitations are also great in some respects [9,10]. For example, polyurethane is usually obtained by reacting diisocyanates or polyisocyanates with alcohols and has poor water resistance due to the presence of a hydrophilic group –CH_2_OH on the molecular chain of the polyurethane [11,12]. The introduction of hydrophobic structure and crosslinked structure into the molecular chain of polyurethane is the main approach to solve the underwater performance of polyurethane. The fluorine segment and silicon segment are the main constitutuents of two hydrophobic structures introduced at this stage [13]. Zhihau Li et al. [14] successfully prepared fluorosilicone polyurethane. The results showed that when the amount of hydroxyl fluorosilicone oil was 40 wt%, compared with polyurethane, the water contact angle of fluorosulfur polyurethane (FSPU) increased to 113.6°, the water absorption rate decreased to 0.7%, and the hydrophobic property was significantly improved. Yi Zhou et al. [15] prepared hydrophobic fluorosilicone polyacrylate polyurethane coating and clarified the surface self-segregation characteristics of the fluorine segment and the silicon segment. The coating showed excellent surface hydrophobicity with amphoteric points, strong thermal stability, and controllable mechanical properties. Lei Chen et al. [16] prepared the triblock copolymer series with a polyurethane (PU) middle block and fluorinated end blocks, poly 2,2,2-trifluoroethyl methacrylate (PTFEMA). Static contact angle tests demonstrate that the triblock copolymer with PTFEMA of 0.03 already attained a much higher value of the water contact angle (95°) than the value (74°) of the PU homopolymer. It has been demonstrated that the addition of very low levels of nanomaterials can provide excellent composite properties to the polyurethane coatings [17,18,19,20]. To improve the wear resistance of polyurethane elastomer under erosive conditions, R. Zhou et al. [21] studied the effect of Al_2_O_3_ content and different silane coupling agents on mechanical properties and erosion wear properties, and prepared polyurethane matrix composites. The results show that with the increase of Al_2_O_3_ particle content, the wear resistance of the composite increases first and then decreases, and the erosion wear mechanism of the composite was discussed. Zhang Min and Chen Yufei et al. [22,23] added nano-alumina material to the polyurethane, and the coating improved corrosion resistance, wear resistance, and thermal stability. There is little research on the preparation of composite coatings by adding nano alumina to fluorinated polyurethane. The synthesis of composite polyurethane coatings with both anti-corrosion, abrasion resistance, and water resistance is a serious problem that needs to be solved today. In this paper, we introduced fluorinated polyurethane-alumina composite coating, which was prepared by grafting an organofluoroalkyl group in a polyurethane segment and adding nano-alumina, which significantly improved the water resistance and wear resistance of the coating.

## 2. Materials and Methods

### 2.1. Materials

Diphenyl-methane-diisocyanate (MDI) was purchased from Sigma-Aldrich, and was industrially pure. Polytetrahydrofuran ether glycol (PTMEG) was purchased from Shanghai kuang chemical material co. LTD (Shanghai, China), molecular weight 1000, vacuum dehydration at 70 °C for 3 h before being used. Perfluorooctyl alcohol (TEOH-8) was provided by Hengtong fluorine chemical co. LTD (Fuxin, China), and had chemical purity of 97%(GC&T) (Gas chromatography and Techincal grade). Triethylene glycol (TEG) was purchased from Sigma-Aldrich, and had chemically purity of 99%(GC&T). Nano-alumina (mean particle diameter = 70.5 nm, specific surface area = 195.59 m^2^·g^−1^) was provided by Zhengzhou Tianma new material co. LTD (Zhengzhou, China). KH-550 was purchased from Hubei huaxin organic silicon new material co. LTD (Hubei, China), and was chemically pure. The solvents used in the experiments were N,N-dimethylformamide (DMF), N,N-dimethylacetamide (DMAC), tetrahydrofuran (THF), methyl ethyl ketone, acetic acid, acetone, and methanol. The above solvents were purchased from Shanghai han photochemical reagent co. LTD (Shanghai, China), and they were all chemically pure.

### 2.2. Experiments Methods

#### 2.2.1. Surface Modification of Nano Alumina

The silane coupling agent KH-550 was dissolved in an ethanol solution, and the pH of the reaction mixture was adjusted to 4-5 using acetic acid and stirred at room temperature for 2 h. Then, the nano-alumina and KH-550 were added to the reaction kettle and stirred at 60 °C for 4 h. KH-550 accounted for 1.5% of the mass of the nano-alumina. The alumina granules obtained by centrifugation were washed several times with ethanol and then dried for use.

#### 2.2.2. Preparation of Fluorinated Polyurethane-Nano-Alumina Composite Coating

**Figure 1:**
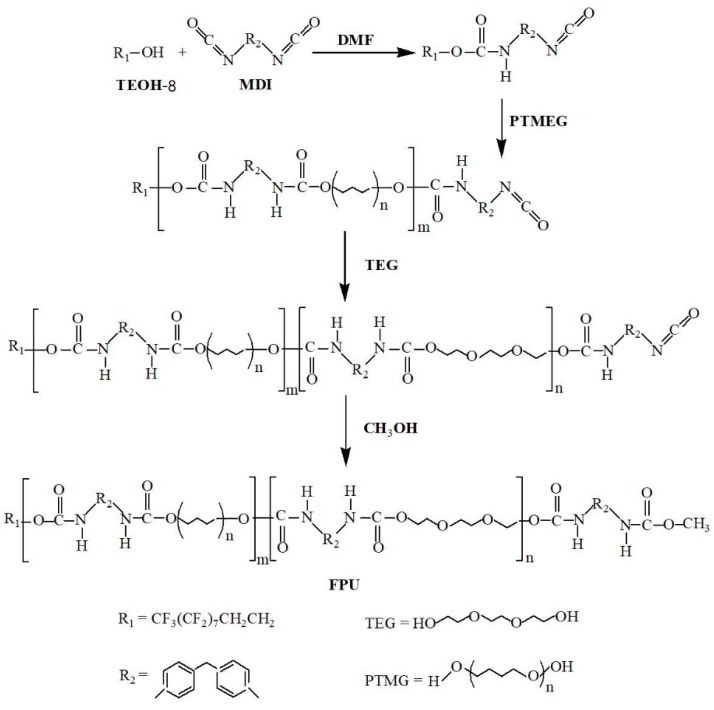
Reaction scheme of FPU (fluorinated polyurethane).

The first step involved dissolving MDI in DMF and pouring it into a four-necked flask protected with nitrogen. The remaining three ports of the four-necked flask were equipped with a stirrer, a thermometer, and a constant pressure dropping funnel. Perfluorooctylethyl alcohol was added dropwise to the DMF solution in the flask at 4d/s using a constant pressure dropping funnel. The reaction was continued at 50 °C for 2.5 h at a stirring speed of 70 rpm. The molar ratio of TEOH-8 to DMF was 1:1 (the molar ratio of TEOH-8/MDI was about 0.5, and the synthesized polyurethane was referred to as FPU-0.5). The four-necked flask was heated to a temperature of 60 °C, polytetrahydrofuran was added to the reaction system, and the mixture was reacted for 2 h using a stirrer. A fluoropolyurethane prepolymer with PTMEG as a soft segment was synthesized in this process. In the second step, nano-alumina was dispersed in acetone at room temperature for 2 h using ultrasonic equipment, which was manufactured by Dongguan taiming CNC machinery co. LTD (Dongguan, China). Additionally, the ultrasonic frequency was set to 50 kHz. The dispersed alumina system was added to the pre-polymer, stirred for 2 h (after being uniformly dissolved), added TEG to the flask, and reacted at 80 °C for 3 h. In the third step, the temperature was adjusted to 70 °C for distillation (after the distillate was significantly reduced), the temperature was further increased to 100 °C, and vacuum distillation was performed by a vacuum pump. The mixture was then cooled to room temperature when the bubbles were no longer generated. Finally, the reaction material was poured on pre-treated alloy steel sheets (surface sandblasted roughened, brushed with epoxy two-component primer, and primer cured for 6 h) and placed in a constant temperature electric oven at 60 °C for curing to fully matured. That is, a fluorinated polyurethane-alumina composite coating having a perfluoro segment on one side and a hydrophobic –COOCH_3_ group on the other end was obtained. FPU synthesis scheme is shown in Figure 1.

Five different alumina content coatings were prepared in this experiment. The mass percentages of alumina were 0%, 1%, 2%, 3%, and 4%, recorded as FPU-0.5, Al_2_O_3_-FPU (1), Al_2_O_3_-FPU (2), Al_2_O_3_-FPU (3), and Al_2_O_3_-FPU (4).

Meanwhile, the experiment was carried out by reacting 1,5-naphthalene diisocyanate(NDI) with Ethylenediamine (EDA) (n(NDI):n(EDA) = 1.25) and NDI with 1,4-butanediol(BDO) (n(NDI):n(BDO) = 1.25) to synthesize the compound as reference compounds.

### 2.3. Characterizations

To visualize whether the FPU was synthesized in this experiment, the ATR-FTIR characterization method was used to detect and analyze the polymerization product. The FPU-0.5 was tested by Nicolet is 10 Fourier transform infrared spectrometer. The ATR mode was selected and the scanning precision was set to 4 cm^−1^. The number of scans was 32 times, and the test range was 400 cm^−1^ to 4000 cm^−1^ [24].

COXEM EM-30AX scanning electron microscope (COXEM Co., Ltd. Daejeon, Korea ) was used to examine the samples with different nano alumina content. The surface of the samples was sprayed with a 10–20 nm gold plating layer by vacuum evaporator [25].

OCA20 video contact angle tester was used for testing (Shanghai Sunzern Instrument Co., Ltd., Shanghai, China). The syringe, dosing tube, and injection needle were thoroughly wetted with deionized water before the experiment, and the syringe was filled with deionized water without any blistering. The water droplets dripped on the surface of the coating for 1 min before observation, and three test points were taken for each sample and averaged.

EMS-10 magnetic stirring heated water bath was used to test (Shanghai Meiyingpu Instrument Manufacturing Co., Ltd., Shanghai, China). The sample with mass m_1_ was weighed, and the area of sample 2/3 was immersed in warm water. The water temperature was set at 30 °C, and the magnetic stirring speed was 240 rpm. After 200 h, the water was removed from the surface with filter paper and the sample denoted as m_2_ was weighed. The water absorption of the sample was calculated as follows:

(1) $$\eta =\left[\left(m_{2}-m_{1}\right)/m_{1}\right]\times 100\%$$

The sample with the original mass of m_1_ was placed in an erosion wear tester, and the wear resistance test was carried out. The sample size was 120 mm × 100 mm × 2mm, and the mortar was used for the wear test. The quartz sand grain size of the mortar was 0.1–0.4 mm, mortar concentration 30%–50%, test line speed was 15 m/s, and the angle formed 45°. The erosion test lasted 480 h, and the mortar was replaced every 24 h during the experiment. After the erosion wear test, the sample was dried and its mass measured as m_2_ by an electronic balance with an accuracy of 0.0001 g. In the experiment, the cavitation wear resistance of the material was evaluated by the actual cavitation amount. When the actual cavitation amount C was smaller than the cavitation guarantee amount C_n_ (C ≤ C_n_) converted according to the actual time, it was regarded as qualified.

According to the actual running time calculation of cavitation erosion quantity, the computation formula is as follows [26]:

(2) $$C_{n}=C_{r}{\left(t_{n}/t_{r}\right)}^{n}$$

(3) $$C_{r}=K_{m}D^{2}$$

In which C_r_ stands for cavitation guarantee amount, kg; n is for conversion index, n = 1.0; K_m_ stands for the quality assurance coefficient, K_m_ = 0.8; D is for the nominal diameter of sample rotation, 100 × 10^−3^ m; t_n_ is for the actual running time, 480 h; and t_r_ is for base running time, 8000 h.

In this test, the sample size was 120 mm × 100 mm × 2 mm, C_r_ = 0.8 × (100 × 10^−3^)^2^ = 8 × 10^−3^ kg. The conditions such as sediment content and impact speed were 5–30 times the actual conditions of hydraulic over-current components, t_n_ ≈ 5 × 480 h, n = 1.0. They were substituted and calculated as follows:

C_n_ = 8 × 10^−3^ × (5 × 480 ÷ 8000)^1^ = 2.4 × 10^−3^ kg.

The actual cavitation amount is calculated as follows:

(4) $$C=m_{1}-m_{2}$$

PosiTest AT pull-off adhesion tester was used to verify the adhesion of the sample before and after the water absorption test and the erosion-resistant test. Three test points were selevted for each sample. The nearest distance between the edge of each two test points was greater than 15 mm, taking the average of the test results.

Mechanical properties were tested at 25 °C with SANS computer controlled electronic tensile testing machine (Lixian Instrument Technology Co., Ltd., Dongguan, China). Three groups of tensile tests were conducted, with elongation rates of 10%, 30%, and 70%, and marked as FPU-0.5(10), FPU-0.5(30), and FPU-0.5(70). The elongated samples were, respectively, fixed on steel sheets and kept for 24 h to prevent them from rebounding, to perform differential scanning calorimetry analysis.

Differential scanning calorimetry (DSC) was carried out using the TA instrument company Modulated DSC-2910 instrument (Shanghai, China). For the first time, heat from room temperature was raised to 200 °C and stabilized for 3mins to eliminate the heat history. Then, it was cooled to −100 °C at 20K/min and stabilized for 5mins. The scanning temperature range of the test was −100–200 °C, and the heating rate was 10 K/min under the atmosphere of nitrogen.

## 3. Results and Discussion

### 3.1. Analyses of FTIR-ATR

**Figure 2:**
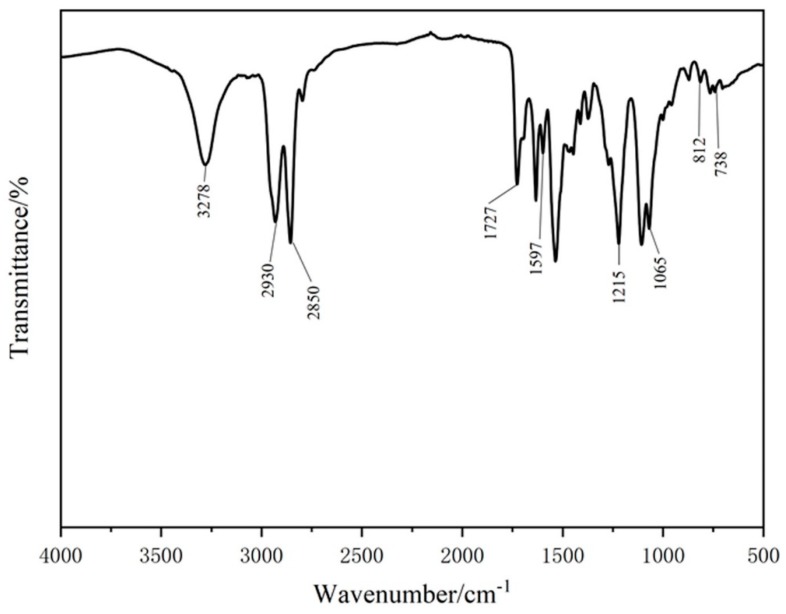
Infrared absorption spectra of FPU-0.5.

Fourier infrared absorption spectrum is shown in Figure 2. As in figure, FPU-0.5 has the characteristic peaks [27,28]: the stretching vibration peak of N–H appears at 3278 cm^−1^. The characteristic peak of 2850–2930 cm^−1^ corresponds to the stretching vibration of the polyether soft segment –CH_3_, =CH_2_, ≡CH, and the peak of 1727 cm^−1^ is the stretching vibration of C=O. 1597 cm^−1^ is the benzene ring skeleton vibration. The disappearance of the absorption peaks of –NCO (2270 cm^−1^) and –OH (3340 cm^−1^) proves the synthesis of FPU. The characteristic peak at 1542 cm^−1^ is the bending vibration of N–H and the stretching vibration of C–N. At 738.20 cm^−1^, 812.17cm^−1^, and 1215.97 cm^−1^, they are, respectively, =CF_2_, –CF_3_, and –CF stretching vibration absorption peaks, which confirms the existence of fluorine element. According to the above analysis, fluorinated polyurethane without hydrophilic –CH_2_OH group can be obtained using the method adopted in this paper.

### 3.2. SEM Analysis

**Figure 3:**
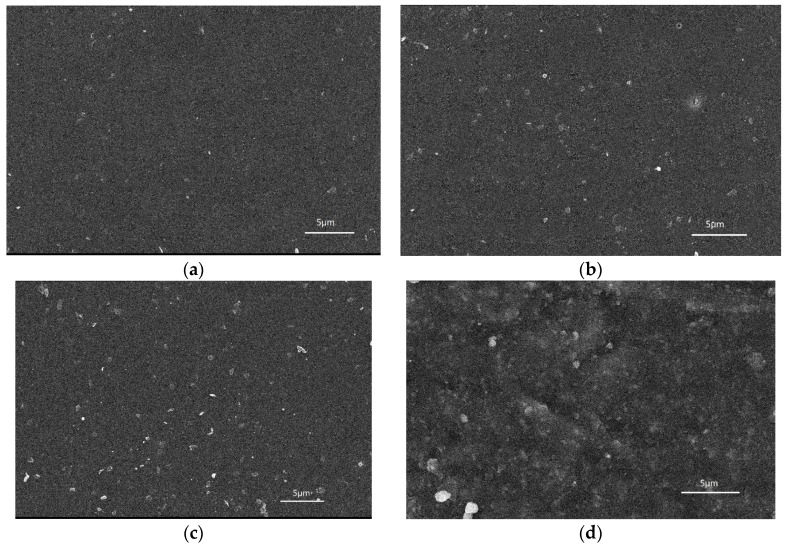
Surface morphology of coatings with different nano-alumina content: (**a**) Al_2_O_3_-FPU(1), (**b**) Al_2_O_3_-FPU(2), (**c**) Al_2_O_3_-FPU(3), and (**d**) Al_2_O_3_-FPU(4).

It can be seen in Figure 3 that as the content of nano-alumina added to the coating material increases, the amount of nano-alumina observed on the surface of the coating increases significantly, while some of the larger structures are due to the agglomeration of the nanoparticles. When the nano-alumina content was 4%, the agglomeration phenomenon drastically increased. When the nano-alumina content was 3%, the dispersion effect was greatest. The fluorocarbon chain with lower surface energy migrates to the surface layer of the coating to form a micro-nano-scale protrusion structure, and the surface roughness becomes large, thereby enhancing the water resistance of the coating. When the coating was subjected to wear, the alumina on the surface of the coating had large hardness and could withstand most of the friction, thus reducing the rate of wear of the coating. The C–F segment with high bond energy near the surface layer has high strength and water resistance, which prevent wear and continue to penetrate the coating [29,30].

### 3.3. Analysis of Water Resistance of Coating

As the amount of alumina increases, the contact angle of water droplets on the surface of the polyurethane coating increases from 95.3° of FPU-0.5 to 123.1° of Al_2_O_3_-FPU (4), while the water absorption of the coating in Table 1 was also significantly reduced from 2.52% to 1.04%. Obviously, the hydrophobic properties of the coating increase with increasing alumina content. It is worth noting that when the alumina content was increased from 3% to 4%, the water contact angle of the coating increased less, and the water resistance did not change significantly.

### 3.4. Analysis of Cavitation Wear Resistance of Coating

The variation of the friction coefficient of FPU-0.5 was relatively large, ranging from 0.38 to 0.45. The fluctuation range of friction coefficient of Al_2_O_3_-FPU (4) was small and stable, ranging from 0.65 to 0.68. The amount of alumina increased slightly, and the friction coefficient of the coating increased gradually. The reason for this is that the alumina protrusion on the surface of the coating increases with the increase of the content. According to the data in Table 2, the actual cavitation amount C of the coating is less than the cavitation guarantee amount C_n_ (C < C_n_ = 2.4 × 10^−3^ kg) converted to the line time, which is the standard for the material cavitation guarantee. In addition, with the increase of alumina content, the amount of cavitation of the coating is also reduced from 2.3755 × 10^−3^ kg of FPU-0.5 to 0.9035 × 10^−3^ kg of Al_2_O_3_-FPU (4), and the anti-wear ability is increased by 61.9% compared with FPU-0.5. The erosion resistance performance is significantly improved. The coefficient of friction of the coating increases, and the amount of cavitation wear decreases. The anomaly of the coating is due to the nano-alumina particles. The addition of nano-alumina increases the surface roughness of the coating and increases the friction coefficient. However, due to the higher hardness, alumina can better resist the impact of abrasive particles and transfer the impact energy to the polyurethane matrix. The coating achieves the effect of rigidity and flexibility, and the overall wear resistance is stronger.

**Figure 4:**
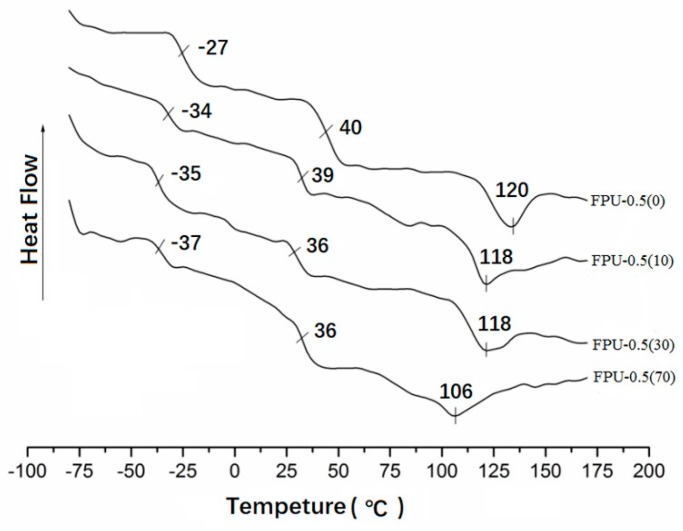
Differential scanning calorimetry (DSC) curves of different elongation FPU-0.5.

In order to further investigate the internal structural changes of the coating during wear, DSC was used for testing. Figure 4 shows the DSC curves of FPU-0.5, and elongations of 0%, 10%, 30%, and 70% are shown in Table 3. The glass transition temperatures of NDI+EDA, NDI +BDO, PBA-2000, and PTMG-3000 were compared as a reference.

Figure 4 shows the DSC curves of FPU-0.5 under different stresses. The temperature range of −27 °C to −37 °C is the glass transition temperature (Tg_(SS)_) of the polyester-polyether soft segment phase, the temperature range of 32 °C–39 °C is the glass transition temperature of the hard segment phase (Tg_(HS)_), and the extreme turning point of the hard segment crystalline melting temperature (Tm) appears in the temperature range of 105 °C–128 °C.

**Figure 5:**
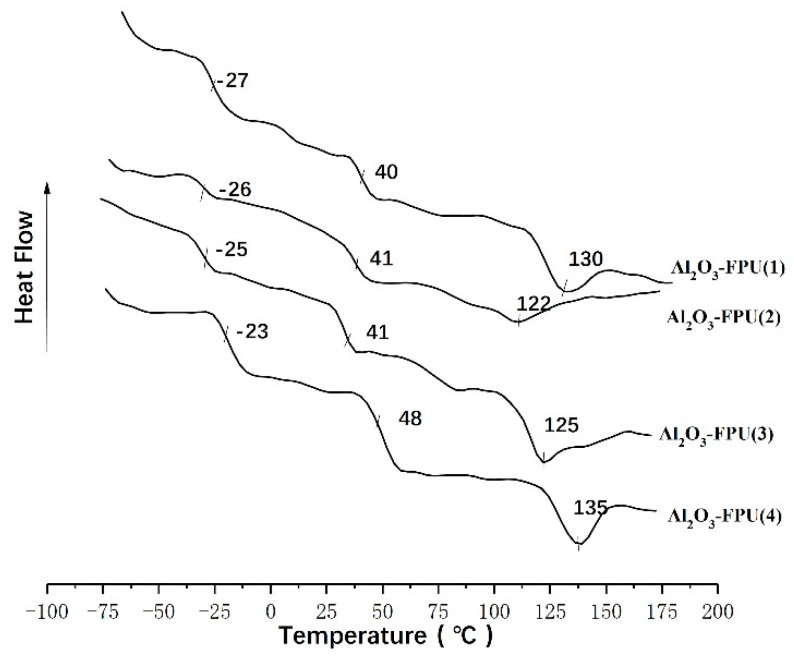
DSC curves of different nano alumina content coatings.

It can be seen from Figure 5 that the glass transition of soft and hard segment of FPU with nano alumina increased, which may have been caused by the introduction of nano alumina. Since the nano-alumina has a strong polarity, when it was introduced into the polyurethane, a large number of hydrogen bonds formed between the soft and hard segments, and the force between the soft and hard segments increased. The degree of microphase separation in the soft and hard sections became smaller, so the glass transition temperature increased.

With the increase of external force, the Tg_(SS)_ and the Tg_(HS)_ of the polyurethane coating and hard segment crystallization temperature Tm of the polyurethane coating decreased. The glass transition temperature of the soft segment of FPU-0.5 exceeded those of the reference compounds, even at the highest external force. It is reported that the hardness of the soft segment phase is higher, the anti-friction performance is better, the glass transition temperature of the hard segment phase is lower, and the hard segment phase is more resistant to impact [31].

For the Tg in the block copolymer, it can be calculated by the Fox equation [32], as follows:

(5) $$\frac{1}{\mathrm{Tg}}=\frac{W_{1}}{Tg_{1}}+\frac{W_{2}}{Tg_{2}}$$

(6) $$\frac{1}{{\mathrm{Tg}}_{\mathrm{SS}}}=\frac{W_{\mathrm{SS}}}{Tg_{1}}+\frac{W_{\mathrm{SS}}}{Tg_{2}}+\frac{W_{\mathrm{HS}}}{Tg_{3}}+\frac{W_{\mathrm{HS}}}{Tg_{4}}$$

(7) $$\frac{1}{{\mathrm{Tg}}_{\mathrm{HS}}}=\frac{W_{\mathrm{SS}}}{Tg_{1}}+\frac{W_{\mathrm{SS}}}{Tg_{2}}+\frac{W_{\mathrm{HS}}}{Tg_{3}}+\frac{W_{\mathrm{HS}}}{Tg_{4}}$$

where Tg is the glass transition temperature Tg_(HS)_ of the hard segment phase or the glass transition temperature Tg_(SS)_ of the soft segment phase under different stresses. W_1_ and W_2_ are, respectively, the mass fraction of the structural units 1, 2 participating in the copolymer in the phase (W_1_ + W_2_ = 1); Tg_1_, Tg_2_ is the crystallization transition temperatures of structural units 1, 2. According to equations (6) and (7), the Tg_(SS)_ and Tg_(HS)_ of the soft and hard segments, and the Tg of the model compound, the microphase separation of the block polyurethane can be calculated as the soft segment unit in the soft and hard segment phase [33]. The mass fractions of the hard segment units are represented by W_SS_ and W_HS_; the results are shown in Table 4.

As the external force of FPU-0.5 increases, the soft fraction structural unit mass fraction W_SS_ decreased from 21% to 12% in the hard segment phase. Additionally, the hard segment unit mass fraction W_HS_ also dropped from 22% to 17% in the soft segment phase of polyurethane. The reason for this change is that the C–N bond between the soft and hard segments breaks in the process of increasing external pressure, resulting in a decrease in the mixed density of the soft and hard segments. When the elongation was small, the C–N bond was not broken, but the hydrogen bond and the subvalent bond may have broken at this moment, improving the degree of microphase separation. At the same time, the cross-linking action of the soft and hard segments was ensured, and the mechanical dynamic synergistic effect between the microscopic phases improved.

**Figure 6:**
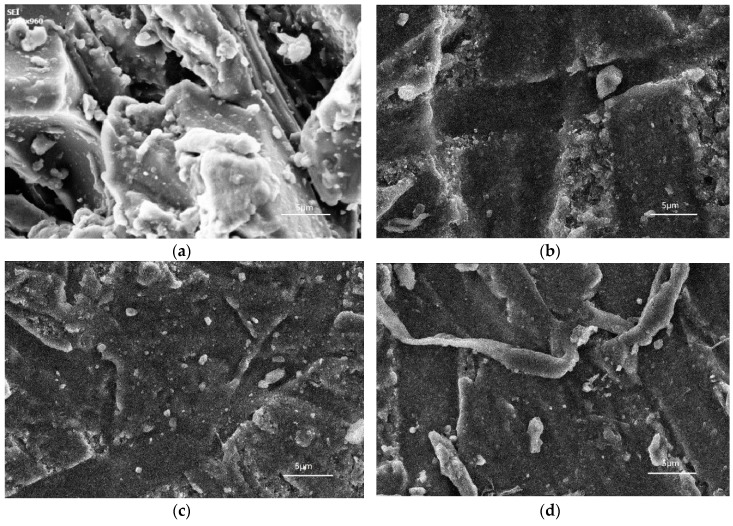
Surface morphology of coatings after wear: (**a**) Al_2_O_3_-FPU(1), (**b**) Al_2_O_3_-FPU(2), (**c**) Al_2_O_3_-FPU(3), and (**d**) Al_2_O_3_-FPU(4).

It can be seen in Figure 6, under the repeated action of wear particles, micro jets, and shock waves, that the macroscopic macromolecular chains are destroyed, and the partially broken flexible segments are separated from the matrix, forming furrows, holes, and other damage on the surface of the coating. The glassy hard segment remains on the surface of the coating, and, due to its high hardness and modulus, the coating surface is prevented from expanding to a greater extent on the coating. The hard segment microdomains increase the intermolecular cohesive energy due to the introduction of higher polarity fluorine groups, thereby increasing the tensile strength and elastic modulus of the hard segment microdomains. When the coating was subjected to erosion, the hard segment on the surface of the coating resisted the wear of the abrasive particles, and the impact energy was transmitted inward through the hard segment to the soft segment of the elastic state inside the coating. The soft segment molecular chain provides buffering and absorption of impact energy, and overall improves the cavitation wear resistance of the coating [34,35,36].

## 4. Conclusions

In this paper, fluorine is successfully introduced by reacting perfluorooctylethyl alcohol (TEOH-8) with diphenylmethane diisocyanate (MDI). Attenuated total reflection-Fourier transform infrared spectroscopy (ATR-FTIR) analysis shows that the FPU synthesized in this experiment does not contain hydrophilic –CH_2_OH groups. Within the experimental range, the water resistance results indicate that the water resistance of the coating increases as the content of nano-alumina increases. The cavitation wear test shows that the actual cavitation amount C of the FPU coating is 0.9035 × 10^−3^ kg, which is less than the cavitation guarantee amount C_n_ according to the running time, and the anti-wear ability is improved by 61.9%. The observation of the coating shows that the high hardness alumina particles on the surface of the coating and the CF chain with high surface bond energy make the coating have high strength and excellent wear resistance and water resistance, which can prevent wear from continuing to penetrate the coating, and the life of the coating is improved. The results show that the alumina-modified fluorinated polyurethane composite coating is significantly improved in terms of water resistance and wear resistance.

## Figures and Tables

**Table 1:** The data of water resistance experiments.

Sample	Contact Angle(°)	Weight, m_1_/g	Cohesive Force σ_1_/MPa	Water Absorption, η/%	Weight, m_2_/g	Cohesive Force σ_2_/MPa
FPU-0.5	95.3	57.3724	12.61	2.52	58.8182	10.44
Al_2_O_3_-FPU(1)	104.2	57.8927	11.23	1.82	58.9463	10.31
Al_2_O_3_-FPU(2)	117.7	59.4325	10.85	1.46	60.3002	10.79
Al_2_O_3_-FPU(3)	122.8	58.4293	9.73	1.08	59.0603	9.68
Al_2_O_3_-FPU(4)	123.1	58.7361	7.17	1.04	59.3470	7.13

m_1_/σ_1_ is the weight/cohesive force of fluorinated polyurethane before the water absorption test; m_2_/σ_2_ is the weight/cohesive force of fluorinated polyurethane after the water absorption test; η = (m_2_ − m_1_)/m_1_ × 100%.

**Table 2:** The data of abrasive resistance experiments.

Sample	Friction Coefficient/μ	Weight, m_1_/g	Weight, m_2_/g	Cavitation Erosion, C/g
FPU-0.5	0.38–0.45	184.7388	182.3633	2.3755
Al_2_O_3_-FPU (1)	0.52–0.58	183.2513	182.0060	1.2453
Al_2_O_3_-FPU (2)	0.58–0.64	183.3752	182.2909	1.0843
Al_2_O_3_-FPU (3)	0.61–0.66	184.4328	183.5015	0.9313
Al_2_O_3_-FPU (4)	0.65–0.68	184.3437	183.4402	0.9035

m_1_/m_2_ is the weight of samples before/after the water absorption test; C = m_1_ − m_2_.

**Table 3:** Results of DSC scanning of polyurethane elastomers with different elongation FPU-0.5.

Sample	Soft Segment Phase	Hard Segment Phase	PU Melting Temperature	Soft Segment Structural Unit		Hard Segment Structural Unit	
T/°C	Tg _(SS)_	Tg _(HS)_	Ta	PBA2000	PTMG3000	NDI+EDA	NDI+BDO
FPU-0.5(0)	−27	40	120	−63	−65	73	74
FPU-0.5(10)	−34	39	118				
FPU-0.5(30)	−35	36	118				
FPU-0.5(70)	−37	36	106				

**Table 4:** Content of structural elements of soft and hard segments in soft and hard regions.

Sample	Hard Phase Region		Soft Phase Region	
	W_HS_%	W_SS_%	W_HS_%	W_SS_%
FPU-0.5(0)	79	21	22	78
FPU-0.5(10)	82	18	21	79
FPU-0.5(30)	84	16	19	81
FPU-0.5(70)	88	12	17	83
